# Age Differences in False Memories Induced by Misinformation: The Role of Attentional Salience of Original Information

**DOI:** 10.1002/pchj.70039

**Published:** 2025-07-12

**Authors:** Ying Guo, Huamao Peng, Bi Zhu

**Affiliations:** ^1^ State Key Laboratory of Cognitive Neuroscience and Learning Beijing Normal University Beijing China; ^2^ Institute of Developmental Psychology Beijing Normal University Beijing China; ^3^ Beijing Key Laboratory of Brain Imaging and Connectomics Beijing Normal University Beijing China; ^4^ IDG/McGovern Institute for Brain Research Beijing Normal University Beijing China

**Keywords:** age differences, attention, false memory, memory consistency, misinformation

## Abstract

People may remember events inaccurately after being exposed to misleading information. This can lead to false memories being reported in multiple interviews. The higher the attentional salience of the original event information (i.e., the extent to which it strongly captures attention during encoding), the less likely young adults are to form false memories. However, it was unknown whether this would also apply to older adults across multiple memory assessments. This study used the misinformation paradigm to examine age differences in memory accuracy and consistency in two recognition tests. It also investigated how attentional salience of the original information influenced memory performances. Thirty young adults (aged 23 ± 2 years) and 30 older adults (aged 70 ± 3 years) saw images of original events, then read misleading narratives, and finally completed a verbal recognition test and a pictorial recognition test based on what they had seen in the original events. Results showed that older adults reported more false memories than young adults in both tests. Older adults were less consistent in reporting true memories across two tests, but there was no age difference in the consistency of false memories. Greater attentional salience helped young and older adults report more original information and less misinformation, though the effect was weaker in older adults. It also helped young and older adults report original information more consistently across tests. Overall, this study showed that how well the original information was encoded significantly influenced eyewitness reports across interviews in young and older adults.

## 1 Introduction

False memories induced by misinformation refer to memory distortions after exposure to misleading information and are explored via the misinformation paradigm in three stages. First, participants see an original event. Next, they are given misleading information about the original event. Third, they are tested on their memory of the original event. Witnesses are often subjected to repeated questioning across various formats. If their answers are inconsistent across tests, it often suggests that their testimony is unreliable. Due to cognitive decline, older adults are often considered vulnerable eyewitnesses who may be more prone to false and inconsistent memory reports. This vulnerability in memory accuracy and consistency may be influenced by the degree of attentional salience of original details. Strong encoding of highly salient original details tends to enhance memory fidelity and reduce both false and inconsistent memories in young adults. However, whether older adults can similarly benefit from the attentional salience of original details remains unclear. Therefore, the present study examined age‐related differences in the accuracy and consistency of memory reports across two different tests between young and older adults, and investigated how the attentional salience of original information affected these differences. This may help to elucidate how the encoding of original information contributes to age differences in memory performance across tests.

### 1.1 Age Differences in Misinformation‐Induced False Memories

Previous studies suggest that older adults are more susceptible to misinformation than young adults (Bulevich and Thomas 2012; Karpel et al. 2001; Mitchell and Johnson 2009; Mueller‐Johnson and Ceci 2004; Roediger and Geraci 2007; Saunders and Jess 2010). For example, a meta‐analysis revealed that older adults aged over 65 years were significantly more prone to misinformation‐induced false memories than young adults were, with a moderate effect size (Wylie et al. 2014). However, there were also studies that either failed to observe an age difference in the misinformation effect or observed a reversed pattern (Huff and Umanath 2018; Tessoulin et al. 2020; West and Stone 2014; Marche et al. 2002). The variability of these findings could be related to different experimental settings during encoding, such as the duration of the to‐be‐remembered episode (Tessoulin et al. 2020; Bulevich and Thomas 2012), the presentation format (West and Stone 2014; Roediger and Geraci 2007), and whether participants were instructed to closely attend to original information for a subsequent memory test (Holliday et al. 2012; Saunders and Jess 2010). These variations in the encoding process may influence the retention of original information, thereby either amplifying or reducing age differences in false memories. To clarify these effects, a systematic exploration of the encoding of original information is needed.

Memory of original information plays a critical role in the emergence of misinformation‐induced false memories. Original memory impairment hypotheses suggest that the loss of original information could render participants more susceptible to post‐event misinformation to fill voids in their original memory representations (McCloskey and Zaragoza 1985; Okado and Stark 2005), or could undermine their ability to employ a clear recollection of the original event to counteract the effects of misleading information (i.e., “recall to reject”) (Jacoby et al. 2001; Brainerd et al. 2003). Successful encoding and retrieval of original information can reduce false memories in young adults, though the effect might be less straightforward in older adults. According to the fuzzy trace theory, older adults have difficulty both in encoding and retrieving precise verbatim traces of original memories (Brainerd and Reyna 2004; Abadie et al. 2021; Greene and Naveh‐Benjamin 2023a). On the one hand, older adults may struggle to engage sufficient attentional resources to encode verbatim representations due to declines in information processing abilities (Greene and Naveh‐Benjamin 2024; Abadie and Guette 2024). On the other hand, despite successful encoding, their verbatim representations of original information remain particularly vulnerable to interference from misinformation (Radvansky et al. 2005; Mitchell and Johnson 2009; Bulevich and Thomas 2012). In both cases, older adults' impaired verbatim recollection of original episodes may lead them to react differently to various encoding conditions and exhibit distinct age‐related patterns of false memories.

Previous research has manipulated the encoding condition of original events and found that encoding influenced age differences in false memories (Marche et al. 2002; West and Stone 2014). For example, Marche et al. (2002) observed that when both young and older adults watched the original slide sequence repeatedly to achieve perfect recall, older adults reported more false memories of misleading details. In contrast, when they only watched a single presentation of the original event, more false memories were reported in young adults than in older adults. This pattern of results may signify that older adults tend to exhibit higher susceptibility to misinformation under strong rather than weak encoding conditions. However, prior studies present two main limitations. First, the specific cognitive processes underlying encoding manipulations (e.g., viewing an event once versus multiple times) remain unclear. Second, these studies typically manipulate the overall encoding of entire episodes, while neglecting the variations among individual details. Specifically, individual details may differ inherently in their attentional salience and the strength with which they are encoded (Saunders 2009). Therefore, this study used a large set of items that varied in attentional salience to examine how encoding‐related attentional differences contribute to age differences in false memories.

### 1.2 Age Differences in Memory Consistency Across Successive Tests

Consistency reflects the correspondence between details provided across multiple retrieval attempts (Rubínová et al. 2022). The examination of memory consistency holds significant practical value because witnesses are often required to recall events across multiple interviews, and inconsistencies between interviews are interpreted as indicators of low credibility (Berman and Cutler 1996; Odinot et al. 2012). Memory consistency is influenced by various factors, including situational and individual‐related variables, but it is also closely tied to memory quality (Henkel 2014a). Studies have shown that the primary cause of memory inconsistency is a lack of specificity and distinctiveness (Price et al. 2016; Rubínová et al. 2022). Memories burdened with interference and confusion due to overlapping information tend to exhibit low consistency. In contrast, highly consistent memories often contain diagnostic details and are accompanied by high confidence and certainty (Henkel 2014a, 2017; Rubínová et al. 2022; Odinot et al. 2012).

Unlike memory accuracy, how young and older adults differ in memory consistency remains relatively underexplored. Only two studies have investigated inconsistencies in older adults' eyewitness reports between successive tests. One study reported that memory changes under interrogative pressure were negatively related to participants' age (18–82 years); however, it did not examine the effect of misinformation (McMurtrie et al. 2012). The other study asked participants to watch a crime video and then answer both misleading and nonleading questions in two successive recognition tests. The results revealed that young and older adults presented similar rates of memory changes in response to both misleading and nonleading questions (Henkel 2014b).

These findings indicate that older adults do not appear to suffer more from inconsistent memory reports than young adults do. However, two limitations are evident in this body of research. First, these studies compared overall memory changes without differentiating between different types of initial responses (i.e., initial true memories that later became false memories vs. initial false memories that later became true memories). Previous research has suggested that true and false memories stem from different mechanisms (Kiat and Belli 2017; Baym and Gonsalves 2010), which contribute differently to memory consistency. True memory consistency is largely driven by the distinctiveness of original information (Price et al. 2016), whereas false memory consistency relies on a firm recollection that misinformation occurred (Wyler and Oswald 2016). This type of false recollection often arises from a highly fluent retrieval of misinformation or from an erroneous integration of misinformation into the original context (Doss et al. 2016). Thus, their consistency should be examined separately to clarify how each memory type persists across tests. Second, these studies administered identical tests and overlooked the fact that, in real life, individuals are often asked to retrieve information repeatedly under different testing conditions, such as by different interviewers (Odinot et al. 2013), in different question formats (Powell and Thomson 1996; Memon and Vartoukian 1996; Krähenbühl et al. 2009), or with different retrieval cues (Gilbert and Fisher 2006). Legal settings specifically require repeated testing in varied formats to avoid confusion and contamination across tests (“Achieving Best Evidence” in the UK, Ministry of Justice 2011). One of the advantages of investigating memory consistency across different testing conditions, rather than identical ones, is that varying retrieval formats more effectively facilitate the recovery of original information (Gilbert and Fisher 2006), and assist in the correction of false memories (Dodson et al. 2015; Bulevich and Thomas 2012; Wyler and Oswald 2016). This approach is particularly promising when the second memory test provides retrieval cues for the original information. For example, studies have shown that older adults' memory accuracy improves when they proceed from recall to recognition (Danckert and Craik 2013), or through stages of a modified cognitive interview (Dodson et al. 2015). The extent of these memory gains between young and older adults remains unclear. Therefore, another aim of this study was to examine how the consistency of true and false memories across tests differed between young and older adults when retrieval cues for the original information were available in the second memory test.

### 1.3 The Effect of Attentional Salience on Eyewitness Memory

Attentional salience of original information refers to its inherent property that dictates the extent to which it captures attention due to its conceptual prominence, perceptual salience, and/or emotional relevance (Pickel et al. 2006). Research on the effect of attentional salience on eyewitness memory has generally supported the idea that increased allocation of attentional resources strengthens memory, particularly for verbatim traces rather than gist traces (Greene and Naveh‐Benjamin 2023b). On the one hand, increased salience of original details helps resist misinformation and reduces the number of false memories reported (Dalton and Daneman 2006; Daneman et al. 2013; Heath and Erickson 1998; Loftus 1979; Luna and Migueles 2009; Mangiulli et al. 2020; Wright and Stroud 1998). For example, Loftus (1979) demonstrated that only 2 out of 46 participants reported false memories for a salient and major detail concerning the central character (i.e., the color of the wallet the thief stole). On the other hand, increased attentional salience enhances memory distinctiveness. Studies have shown that, in young adults, true memories of high‐salience details are reported with greater confidence and often include more contextual information than memories of low‐salience details (Luna and Migueles 2009; Paz‐Alonso and Goodman 2008; Kafkas and Montaldi 2011). These strongly encoded details are more easily distinguished from other overlapping information (Fandakova et al. 2020). In sum, increased attentional salience of original information influences both the quantity and quality of eyewitness memory reports in young adults, which in turn affects the accuracy and consistency of memory reports across tests.

Nevertheless, studies examining how attentional salience of original information interacts with age to influence the accuracy and consistency of misinformation memory reports are rare. Only two studies have investigated its impact on memory accuracy in young and older adults. One study reported that older adults were more susceptible to misinformation for both high‐ and low‐salience details (Saunders and Jess 2010). The other study revealed that older adults reported more false memories for low‐salience details but not for high‐salience ones (Daneman et al. 2013). However, the ceiling effect observed with low‐salience items (both groups reported over 90% false memories) made it difficult to draw clear conclusions. Regarding the effect of attentional salience on memory consistency, only one study focusing on young adults was identified (Wyler and Oswald 2016). The findings revealed that false memories for high‐salience items persisted from the recognition test to the source test, whereas errors related to low‐salience items were significantly reduced. Older adults were not included in this study. In summary, how attentional salience of original information affects age differences in memory accuracy and consistency remains unclear.

### 1.4 The Present Study

The present study had two aims: (1) to examine whether young and older adults differ in the number of true or false memories reported, as well as the consistency of these memory reports across successive tests; (2) to investigate how attentional salience of original information influences these aging effects.

**FIGURE 1:**
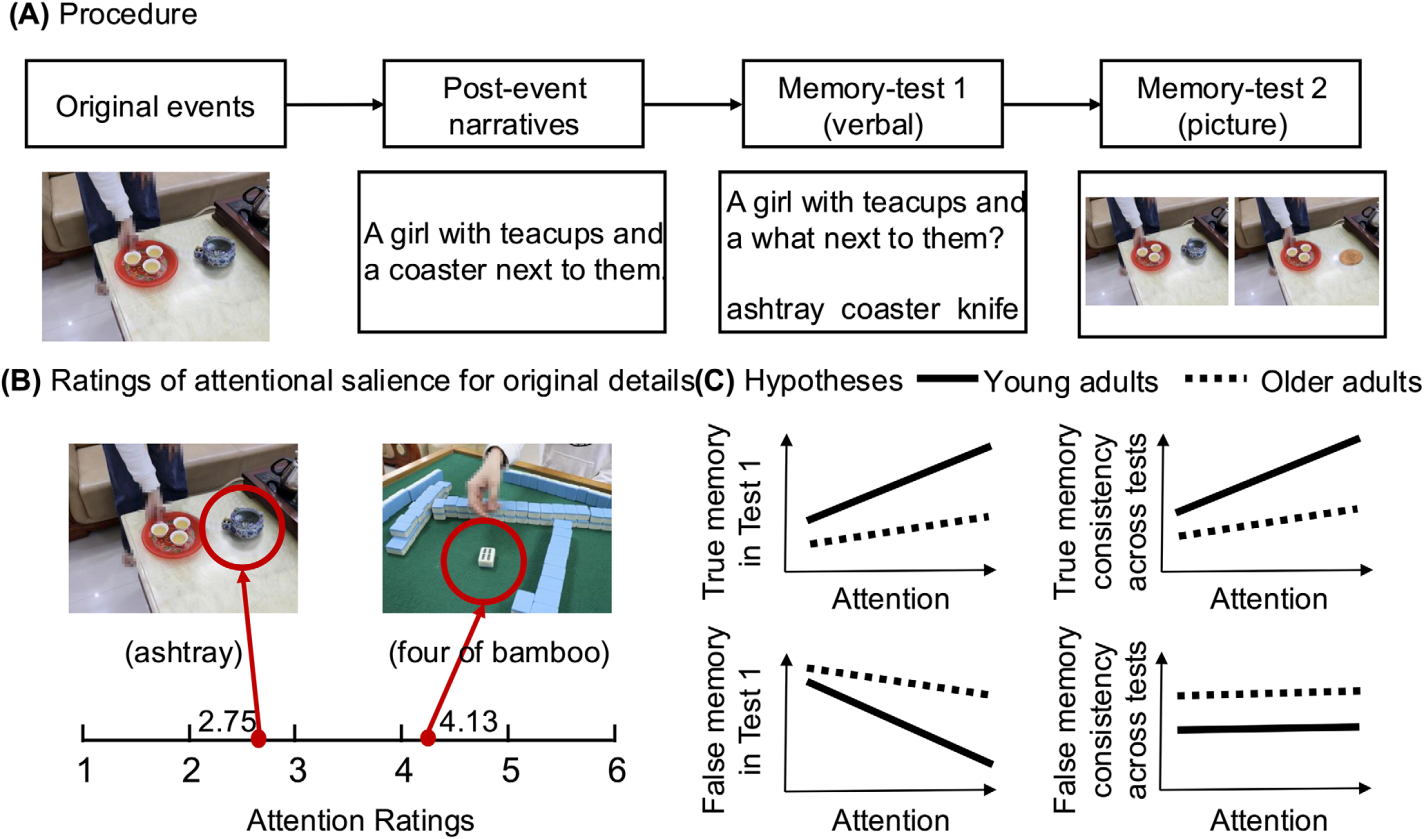
Schematic diagram of the experimental procedure (A), examples of attentional salience ratings for original image details (B), and study hypotheses (C).

To address these aims, the main experiment employed the following procedure (Figure 1A): participants first viewed event images and then read misleading narratives. Two successive tests were subsequently administered to assess their memory of the original event. The first memory test aimed to provide the most reliable measure of memory accuracy in repeated interviews (Steblay and Dysart 2016), and therefore a canonical verbal recognition test was used (Okado and Stark 2005). The second memory test was intended to offer participants an opportunity to correct their initial false memories; thus, it was designed to provide rich retrieval cues for the original information. Specifically, it was a pictorial recognition test embedded within the chronological presentation of the original images. This design follows the principles of the modified cognitive interview to evoke mental reinstatement of original context and facilitate focused retrieval (Dodson et al. 2015). There was a one‐hour interval between the original event and post‐event stage to allow memory consolidation before the introduction of misinformation. This setting also enhances ecological validity, as it reflects situations where witnesses are often misled sometime after the event has occurred. The memory tests were conducted immediately and consecutively to simulate the rapid, successive interviews often employed in legal contexts (Hope et al. 2011).

Attentional salience of original details was assessed through a pilot study in which a separate group of young and older adults rated the extent to which specific image details attracted their attention during the initial viewing period (Christianson and Loftus 1991; Compo et al. 2012; Peace and Constantin 2016) (Figure 1B). An independent group of raters was used for two reasons: (1) the concept of attentional salience reflects inherent properties of the stimulus and is assumed to generalize across individuals; and (2) to avoid contamination of the ratings by exposure to the main experimental procedures—such as misinformation or repeated presentation of the original stimuli—had the same participants been involved.

This study focused on two key aspects of memory performance: memory accuracy and memory consistency. Memory accuracy was indexed by the absolute endorsement rates of true and false memories for misleading items in Test 1. Control items without misinformation were used as a baseline. Memory consistency was assessed by calculating the relative consistency ratios of true and false memories for misleading items across tests. These ratios were defined as the conditional probabilities of retaining true or false memories in Test 2, given that they had already been reported in Test 1 (Zhu et al. 2012). This measure is particularly useful when memory responses are sequential (Dodson et al. 2007), when effects depend on initial memory conditions (Roediger et al. 1996), and when developmental groups with different baseline performance are compared (Danckert and Craik 2013). Given prior research suggesting that overlapping memory traces from misleading items contribute to inconsistency between tests (Price et al. 2016), this study focused exclusively on the consistency of true and false memories for misleading items.

The following hypotheses were proposed (Figure 1C): (1) Older adults should report fewer true memories and more false memories than young adults did. (2) Older adults should be less consistent in reporting true memories but more consistent in reporting false memories across tests than young adults. This hypothesis is informed by findings on age differences in memory quality: older adults' true memories often lack detail and differentiation (Koen et al. 2020), yet they tend to hold false memories with strong confidence (Dodson et al. 2007, 2015), making their false memories more likely to persist across tests. (3) Original details with higher attentional salience should elicit more true memories and fewer false memories in young adults. However, this effect may be less pronounced in older adults due to their difficulty in leveraging stronger encoding of original information to improve memory accuracy (West and Stone 2014; Marche et al. 2002). (4) Original details with higher attentional salience should lead to higher consistency in true memory reports among young adults, though this effect may be less pronounced in older adults. Attentional salience of original details may have minimal impact on false memory consistency in both young and older adults, as it is not directly related to the formation of false recollection that sustains false memories across tests (Wyler and Oswald 2016; Doss et al. 2016).

## 2 Methods

### 2.1 Participants

A power analysis was conducted using G*Power 3.1.9.7 to determine appropriate sample sizes for each group (Faul et al. 2007). Based on a meta‐analysis of 1415 older adults and 2119 young adults, age differences in misinformation‐induced false memories had a moderate effect size (Wylie et al. 2014). Therefore, the power analysis specified an 80% power level, an alpha of 0.05 (two‐tailed), and a medium effect size of 0.25, and results showed that a minimum of 34 participants in total was required. To ensure sufficient power, 30 young adults (18–25 years) and 34 older adults (64–76 years) were enrolled in this study.

Young adults were college students in Beijing, and older adults were residents from neighboring communities. To ensure that participants had no trouble understanding and remembering event materials, all participants had to achieve at least 50% correct memory responses on control questions in Test 1. Three older adults failed to meet the standard and were thus excluded from further analyses. One older adult dropped out before completing all the assessments. The final sample consisted of 30 young adults (21 females and 9 males, mean age = 22.89 years, SD = 1.53) and 30 older adults (21 females and 9 males, mean age = 70.05 years, SD = 3.20). Young adults had an average of 16.77 years of education (SD = 1.28), while older adults had significantly fewer years of education at 13.97 years (SD = 2.20) (*t*(47) = 6.02, *p* < 0.001). The Mini‐Mental State Exam was administered exclusively to older adults to rule out potential cognitive impairment, and all older adults scored above 24 (*M* = 28.30, SD = 1.26) (Folstein et al. 1975). All participants were right‐handed Chinese, had normal or corrected‐to‐normal vision, and had no history of psychiatric or neurological diseases. Written informed consent was obtained from all participants. This study was approved by the Institutional Review Board of State Key Lab of Cognitive Neuroscience and Learning at Beijing Normal University, China.

### 2.2 Materials

The experimental materials consisted of original‐event images, post‐event narratives, verbal and pictorial recognition tests for six events (i.e., gamble, steal, scalper, robbery, loan, and conflicts).

#### 2.2.1 Image and Narrative Materials

Each event included 50 images and 50 narratives. For each event, there were 16 misleading items, 10 control items, and 24 generic items. Misleading items referred to items with contradictory details between images and narratives. Two alternative versions of each misleading item were developed and counterbalanced across participants. For example, one participant saw an image depicting a girl putting three cups of tea on a tray with an ashtray next to it, and later read a narrative stating that a girl put three cups of tea on a tray with a coaster next to it. Another participant saw an image depicting a girl putting three cups of tea on a tray with a coaster next to it, and later read a narrative stating that a girl put three cups of tea on a tray with an ashtray next to it. Control items referred to items with consistent details between images and narratives. For generic items, narratives provided general descriptions of images and were not tested.

#### 2.2.2 Verbal and Pictorial Test Materials

The first memory test assessed original memory for 16 misleading items and 10 control items per event. Each item consisted of a question and three options. For each misleading item, the question was rephrased from the narrative (e.g., “The girl put three cups of tea on a tray next to what?”). The options were verbal descriptions of (1) the detail seen in the original‐event image (e.g., “ashtray”), (2) the detail read in the post‐event misleading narrative (e.g., “coaster”), and (3) a foil detail not present in either the image or the narrative (e.g., “knife”). For misleading items, the endorsement rates of these three options were indicative of true memory, false memory, and foil memory, respectively. For each control item, the question was also rephrased from the narrative (e.g., “What was on the living room wall as the boy turned away?”). The options were verbal descriptions of (1) the detail present in both the original image and the post‐event narrative (e.g., “intercom”), (2) a foil detail not present in either (e.g., “screen”), and (3) another foil detail not present in either (e.g., “camera”). For control items, the endorsement rate of the original option was indicative of correct memory, whereas the averaged endorsement rate of the two foil options was indicative of incorrect memory.

The second memory test assessed original memory for 16 misleading items per event. There were no explicit questions; instead, the chronical presentation of images for control and generic items hinted that the next item was being probed. For each misleading item, the options were two images: (1) the originally seen image (e.g., a girl put three cups of tea on a tray with an ashtray next to it), (2) the other version of this image (e.g., a girl put three cups of tea on a tray with a coaster next to it). The endorsement rate of the original image indicated true memory, whereas that of the other image indicated false memory.

#### 2.2.3 Ratings of Attentional Salience of Image Materials

**FIGURE A1:**
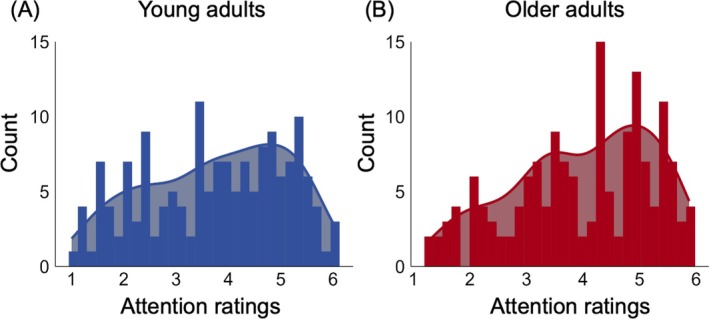
Histograms and density curves of attention ratings for young adults (A) and older adults (B).

The ratings of attentional salience of original details were obtained through a pilot study. Specifically, an independent group of young adults and older adults viewed images from six events sequentially and were then asked to rate all misleading and control items on a scale from one to six. They evaluated the extent to which each specific detail in the original images (e.g., astray) attracted their attention. There were no systematic differences in how young and older raters evaluated the attentional salience of items, and all raters demonstrated an excellent interrater reliability of 0.91 (95% CI [0.89, 0.93]). Therefore, the average rating of all raters was used to signify the attentional salience of each item. Examples of attention ratings for misleading items are shown in Figure 1B. The detailed rating procedure and assessment methods are provided in Appendix A and Figure A1.

### 2.3 Procedure

The experimental procedure is illustrated in Figure 1A. The experiment comprised four stages: the original‐event stage, the post‐event stage, and the two test stages. There was a one‐hour break between the original‐event and post‐event stages and a 10‐min break between the post‐event stage and Test 1. Test 2 was administered immediately after Test 1.

In the original‐event stage, participants were required to view and memorize images from each event. Each event began with the presentation of the event title for 3.5 s (e.g., “gamble”), followed by the presentation of 50 images, each appearing for 4.5 s. The order of events and the version of misleading items were randomized across participants. In the post‐event stage, participants were required to read narratives from each event. They were informed that these narratives came from another participant in the study and were not warned that there might be contradictory details between the images and narratives. The order of events was randomized across participants to ensure different event sequences were adopted in the post‐event and original stages. The rest of the procedure was the same as that used during the original‐event stage.

In Test 1, participants were instructed to answer questions based on the images they had seen during the original‐event stage. Test 1 was a three‐alternative forced‐choice verbal recognition test and was used to evaluate susceptibility to misinformation‐induced false memories. Each event began with the presentation of the event title for 9.5 s. Then, 16 misleading items and 10 control items in each event appeared sequentially. Each question was displayed for 3.5 s, followed by the three options for 3 s. To ensure careful consideration, participants were not allowed to respond during this time and could answer at their own pace once a black border appeared around the options. The order of events, the sequence of questions within each event, and the position of the three options for each question (i.e., left, center, right of the screen) were all randomized across participants.

Test 2 was a two‐alternative forced‐choice pictorial recognition test and was used to examine the consistency of memory reports across the two tests. To investigate memory consistency in the presence of misinformation, only misleading items were retested. All 50 images from each event were presented sequentially, in the same order and duration as during the original‐event stage. For generic and control items, each image was displayed at the center of the screen for viewing. For misleading items, the two image options were displayed side by side, and participants were asked to select the one they had originally seen at their own pace. The order of events was the same as in the original‐event stage, but the position of image options (i.e., left and right sides of the screen) was randomized across participants.

### 2.4 Analyses

The present study examined age differences in memory accuracy and consistency across two tests and investigated how attentional salience of original details influenced memory accuracy and consistency within each age group. Accordingly, four main analyses were conducted: (1) the effect of age on memory accuracy, (2) the effect of age on memory consistency, (3) the interaction of age and attentional salience on memory accuracy, and (4) the interaction of age and attentional salience on memory consistency. Memory accuracy was primarily assessed via the proportions of responses in Test 1. Memory consistency, on the other hand, focused specifically on the consistency ratios of true and false memories across tests. Results for these key dependent variables were FDR‐corrected to mitigate the risk of Type I errors. Each analysis was conducted as follows:

First, in examining age differences in memory accuracy, memory responses from Test 1 were the primary focus, as previous guidelines suggest that the first memory test provides the most reliable measure of accuracy in repeated interviews (Steblay and Dysart 2016). A 2 (age group: young and older adults) × 4 (memory type: true, false, foil, and correct) mixed‐effects ANOVA was conducted on endorsement rates in Test 1. Age group was a between‐subjects factor, and memory type was a within‐subjects factor. Endorsement rates were calculated as the ratio of true, false, and foil memory endorsements to the total number of misleading items, and correct memory endorsements to the total number of control items within each participant. To further explore whether older adults were more susceptible to misinformation interference, endorsement rates of memory errors on misleading and control items were compared using a 2 (age group: young and older adults) × 2 (memory type: false and incorrect) mixed‐effects ANOVA.

As a sensitivity analysis, years of education for each participant were included as a covariate to control for the compounding effect of educational attainment (Appendix B). Memory accuracy in Test 2 was also analyzed for completeness (Appendix C). An additional supplementary analysis investigated whether Test 2 improved memory accuracy on misleading items across repeated testing. The detailed methodology for this analysis is provided in Appendix D.

Second, in examining age differences in memory consistency across tests, consistency ratios for true and false memories were computed separately among young and older adults. These ratios were calculated as conditional probabilities by dividing the number of consistent endorsements of true or false memories across tests by the initial endorsements of true or false memories in Test 1, respectively. This approach is useful for comparing successive memory responses when initial performance differs between groups (i.e., young and older adults exhibited different amounts of true and false memory endorsements in Test 1) (Dodson et al. 2007; Danckert and Craik 2013). Specifically, a 2 (age group: young and older adults) × 2 (memory type: true and false) mixed‐effects ANOVA on consistency ratios was conducted. Years of education were added as a covariate in the sensitivity analysis (Appendix E).

Third, to assess the interaction between age and attentional salience on memory accuracy, multiple linear regression analyses were conducted on an item‐by‐item basis. Three separate models were constructed to predict the proportions of participants reporting: (1) true memories for each misleading item, (2) false memories for each misleading item, and (3) incorrect memories for each control item in Test 1. Each model included three predictors: (1) the attention rating of each item, (2) age group (young adults coded as 0 and older adults coded as 1), and (3) their interaction. FDR correction was applied across the three models in the presence of significant predictions.

Since young and older adults were imbalanced in terms of years of education, sensitivity analyses were conducted on a subset of older adults with education levels matched to those of young adults. The methodology for this analysis is detailed in Appendix F. For completeness, the effects of age and attentional salience on memory accuracy in Test 2 were also examined and are reported in Appendix G.

Fourth, to evaluate the interaction between age and attentional salience on memory consistency across tests, two multiple linear regression models were constructed for the consistency ratios of true and false memories. The ratios were calculated by dividing the number of participants who consistently reported true or false memories for a misleading item across tests by those who initially reported true or false memories for that item in Test 1. Attention ratings, age group, and their interaction were included as predictors. FDR correction was applied across the true and false memory models in the presence of significant predictions. Additionally, sensitivity analyses that included young and older adults with matched education levels are reported in Appendix H.

## 3 Results

### 3.1 Age Differences in Memory Accuracy

**FIGURE 2:**
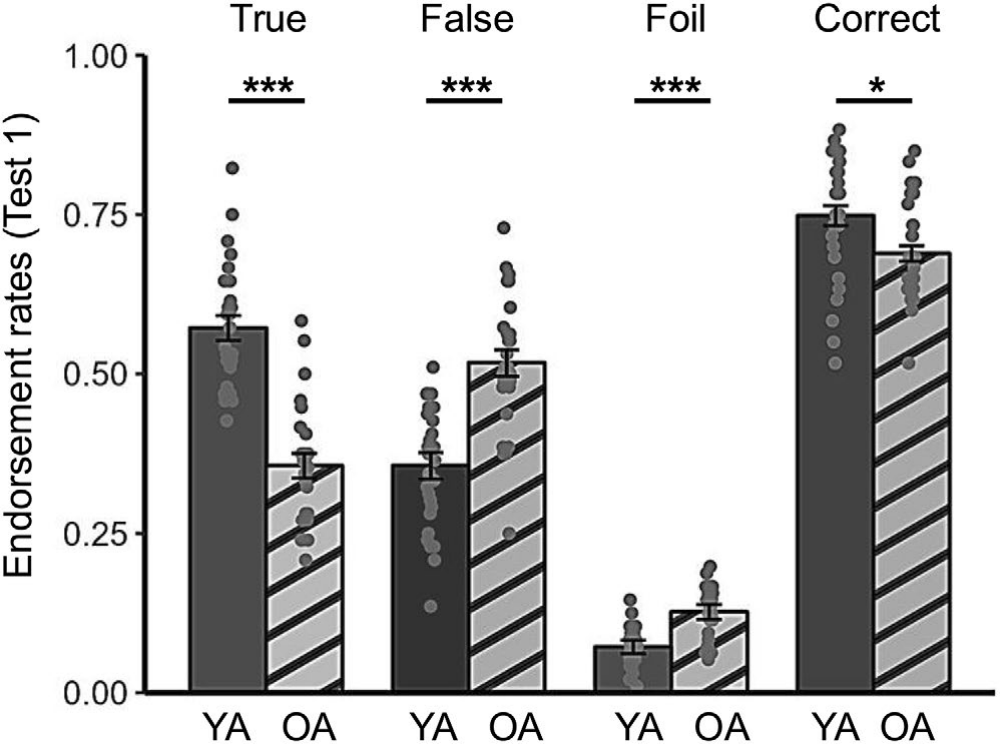
Endorsement rates of true, false, foil, and correct memories in young adults (YA) and older adults (OA) in Test 1. Each dot represents one participant's response. The error bars represent the within‐standard errors of the mean. *p < 0.05; ***p < 0.001.

The endorsement rates for each memory type in Test 1 among young and older adults are shown in Table 1. The 2 (age group: young and older adults) × 4 (memory type: true, false, foil, and correct) mixed‐effects ANOVA revealed a significant interaction between age group and memory type, *F*(3,174) = 45.80, *p* < 0.001, *η*_*p*_^2^ = 0.44 (Figure 2). Compared with young adults, older adults had lower rates of true memories (*t*(58) = −9.05, *p* < 0.001, Cohen's *d* = −2.34), higher rates of false memories (*t*(58) = 6.33, *p* < 0.001, Cohen's *d* = 1.63), higher rates of foil memories (*t*(58) = 5.68, *p* < 0.001, Cohen's *d* = 1.47), and lower rates of correct memories (*t*(58) = −2.58, *p =* 0.012, Cohen's *d* = −0.67). The inclusion of education as a covariate yielded similar results, except that age differences in the endorsement rate of correct memories were not statistically significant (Appendix B).

**TABLE 1:** Means and standard deviations of endorsement rates for memory responses among young and older adults in Test 1.

Age group	True	False	Foil	Correct	Incorrect
Young	0.57 (0.09)	0.36 (0.10)	0.07 (0.03)	0.75 (0.10)	0.13 (0.05)
Older	0.36 (0.09)	0.52 (0.10)	0.13 (0.04)	0.69 (0.08)	0.16 (0.04)

*Note*: Endorsement rates for true, false, and foil memories were calculated by dividing the number of corresponding endorsements by the total number of misleading items. Endorsement rates for correct and incorrect memories were calculated by dividing the number of correct endorsements and the mean number of incorrect endorsements by the total number of control items.

The 2 (age group: young and older adults) × 2 (memory type: false and incorrect) mixed‐effects ANOVA revealed a significant interaction (*F*(1,58) = 20.58, *p <* 0.001, *η*_*p*_^2^ = 0.26), and significant main effects of age group (*F*(1,58) = 50.32, *p* < 0.001, *η*_*p*_^2^ = 0.47) and memory type (*F*(1,58) = 419.77, *p* < 0.001, *η*_*p*_^2^ = 0.88), respectively. Older adults exhibited greater susceptibility to misinformation than young adults even after controlling for their general memory decline (difference between false memory and incorrect memory, older adults: *M*_diff_ = 0.36, *t*(58) = 17.70, *p <* 0.001, Cohen's *d* = 3.11; young adults: *M*_diff_ = 0.23, *t*(58) = 11.28, *p <* 0.001, Cohen's *d* = 2.14). When education was included as a covariate, similar results were observed (Appendix B).

Supplementary analyses further showed that although Test 2 improved memory accuracy relative to Test 1, older adults still reported more false memories than young adults in Test 2. Detailed results of these analyses are presented in Appendix C and D. In summary, the results unanimously showed that older adults were more susceptible to misinformation than young adults, even after controlling for their general memory decline.

### 3.2 Age Differences in Memory Consistency

The 2 (age group: young and older adults) × 2 (memory type: true and false) mixed‐effects ANOVA on consistency ratios revealed a significant interaction effect (*F*(1,58) = 11.28, *p =* 0.001, *η*_*p*_^2^ = 0.16), and significant main effects of age group (*F*(1,58) = 4.67, *p =* 0.035, *η*_*p*_^2^ = 0.07) and memory type (*F*(1,58) = 122.41, *p <* 0.001, *η*_*p*_^2^ = 0.68), respectively. Post hoc analysis revealed that older adults had lower consistency ratios for true memories compared to young adults (older adults: *M*_true_ = 0.81, young adults: *M*_true_ = 0.91, *t*(58) = −5.08, *p* < 0.001, Cohen's *d* = −1.31), while there was no significant difference in consistency ratios for false memories between the two groups (older adults: *M*_false_ = 0.66, young adults: *M*_false_ = 0.64, *t*(58) = −0.85, *p* = 0.399). The results remained the same, except that the main effect of age group was marginally significant after controlling for education (Appendix E).

### 3.3 The Effects of Age and Attentional Salience on Memory Accuracy

**FIGURE 3:**
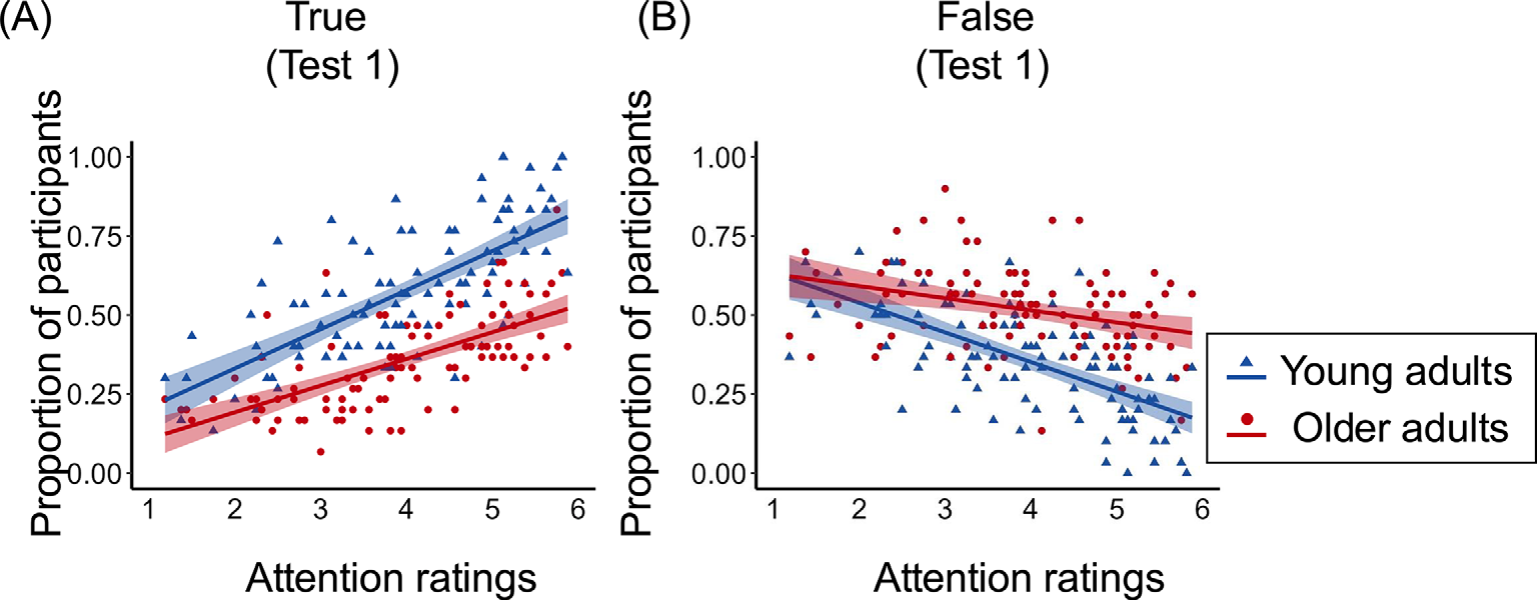
Relationships between attention ratings and the proportions of participants who reported true memories (A) and false memories (B) in Test 1, by age group. Each blue triangle or red dot represents performance on each item in Test 1 for young and older adults, respectively.

The regression analysis examining the effects of attentional salience, age group, and their interaction on true and false memories in Test 1 revealed similar patterns, but in opposite directions. Specifically, a significant interaction was observed for true memories (*β* = −0.04, *t* = −2.48, *p* = 0.014, *p*_FDR_ = 0.021), showing that the attentional salience of original details increased true memories in both age groups, though the effect was weaker in older adults (older adults: *β* = 0.08, *t* = 8.43, *p* < 0.001, *R* ^2^ = 0.43; young adults: *β* = 0.12, *t* = 10.05, *p* < 0.001, *R* ^2^ = 0.52) (Figure 3A). Similarly, a significant interaction was observed for false memories (*β* = 0.06, *t* = 3.49, *p* < 0.001, *p*_FDR_ = 0.002), showing that attentional salience reduced false memories in both age groups, though the effect was weaker in older adults (older adults: *β* = −0.04, *t* = −3.39, *p* = 0.001, *R* ^2^ = 0.11; young adults: *β* = −0.09, *t* = −8.42, *p* < 0.001, *R* ^2^ = 0.43) (Figure 3B). The regression on incorrect memories showed no significant interaction between attentional salience and age group (*β* = 0.01, *t* = 0.79, *p* = 0.431, *p*_FDR_ = 0.431), and the magnitude of the negative relationship between attentional salience and incorrect memories was similar across age groups (older adults: *β* = −0.02, *t* = −2.27, *p* = 0.027, *R* ^2^ = 0.08; young adults: *β* = −0.03, *t* = −3.46, *p* = 0.001, *R* ^2^ = 0.17). The results remained the same when young and older adults with matched education were included (Appendix F). Similar results were obtained for the effects on false memories in Test 2 (Appendix G).

Overall, results from both tests showed that increased attentional salience of original details effectively enhanced true memories and reduced false memories in both age groups. However, the effect was less pronounced in older adults compared to young adults. This inefficiency was specific to the misleading condition, as both groups showed comparable abilities to reduce incorrect memories in the absence of misinformation.

### 3.4 The Effects of Age and Attentional Salience on Memory Consistency

**FIGURE 4:**
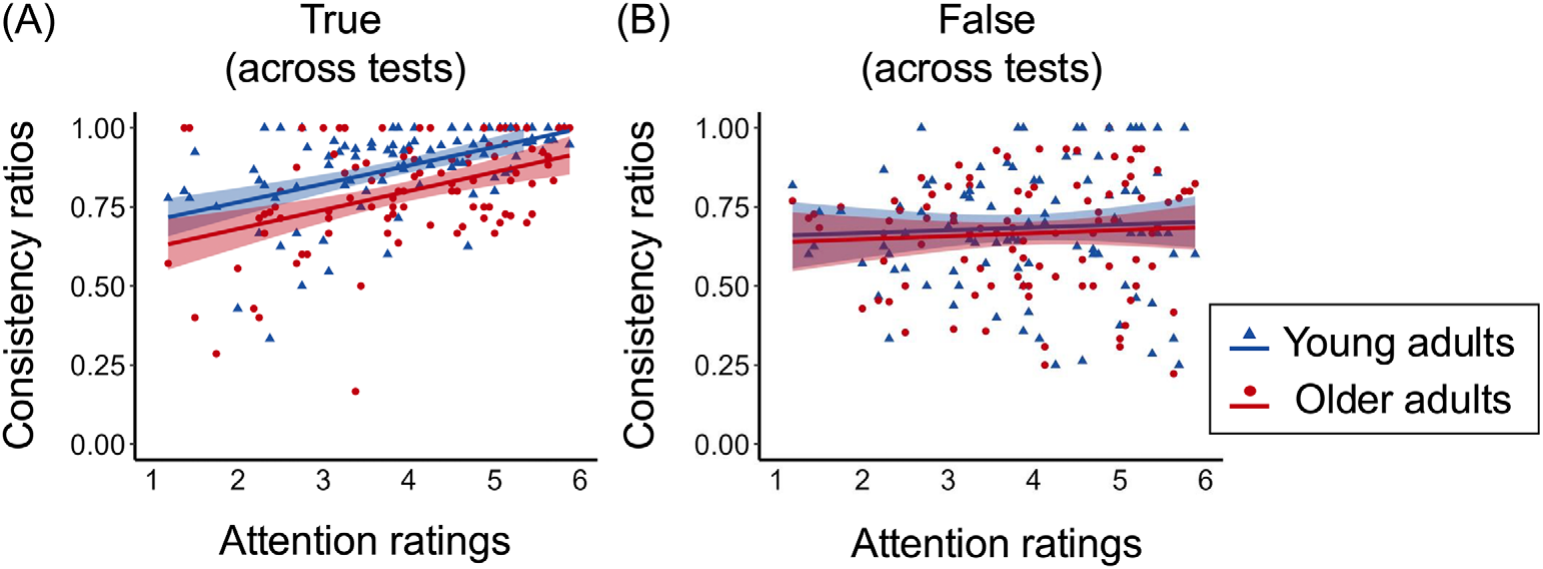
Relationships between attention ratings and consistency ratios of true memories (A) and false memories (B) across tests, by age group. Each blue triangle or red dot represents consistency ratio on each item across tests for young and older adults, respectively.

The regression of attentional salience, age group, and their interaction on the consistency ratios of true and false memories revealed distinct patterns. Results showed significant main effects of age group and attentional salience on true memory consistency (*p* < 0.001), with no significant interaction (*p* = 0.925) (Figure 4A). Attentional salience of original details increased true memory consistency in both groups (older adults: *β* = 0.06, *t* = 4.45, *p <* 0.001, *R* ^2^ = 0.17; young adults: *β* = 0.06, *t* = 5.79, *p <* 0.001, *R* ^2^ = 0.26). For false memory consistency, no significant effects were observed for attentional salience, age group, or their interaction (*ps* > 0.507) (Figure 4B). Similar results were obtained when using young and older adults with matched education. These findings highlight the importance of attentional allocation during encoding in enhancing true memory consistency across age groups, while its effect on false memory consistency was minimal.

## 4 Discussion

Four main findings emerged from the current study. (1) Older adults reported fewer true memories and more false memories than young adults did. (2) Older adults were less consistent in reporting true memories than young adults; however, both groups showed similar levels of consistency in false memories across tests, even when the second test provided retrieval cues for the original information. (3) Attentional salience of original information improved true memories and reduced false memories in both groups; however, the effect was less pronounced in older adults compared to young adults. (4) Attentional salience also improved the consistency of true memories, but had minimal effect on the consistency of false memories in both groups.

### 4.1 Older Adults Were More Susceptible to Misinformation

Consistent with our first hypothesis, older adults reported fewer true memories and more false memories than young adults did in both tests. Test 1 required participants to select the verbal description of original details. The mismatch between this verbal format and the visual encoding of original images may exacerbate older adults' difficulty in verbatim retrieval processes (Brainerd and Reyna 2004). This increased susceptibility of older adults in the verbal recognition test aligns with previous studies that have found older adults struggle to spontaneously recollect details of original events (Roediger and Geraci 2007).

In contrast to Test 1, Test 2 was designed to provide retrieval cues for original images. The effectiveness of Test 2 in reducing false memories was supported by the finding that both groups corrected false memories when they proceeded from Test 1 to Test 2. The recovery of original details in Test 2 may be attributed to the re‐presentation of original images (Guerin et al. 2012). Furthermore, embedding these images within the original context may serve as a scaffold for memory retrieval (Diamond and Levine 2020). However, despite this retrieval support, older adults still reported more false memories than young adults did in Test 2. Whether the observed age differences reflect that older adults are more prone to false memories even with adequate retrieval cues, or whether they were influenced by a carryover effect or a confounding sequence effect due to the fixed order of the two tests could not be determined in this experiment. Future studies should counterbalance the order of the tests and directly compare young and older adults in these two testing conditions.

### 4.2 Older Adults Were Less Consistent in True Memories, but Not False Memories

Distinct age‐related patterns were observed in the consistency of true and false memories. This finding supports the notion that true and false memories stem from different mechanisms (e.g., Baym and Gonsalves 2010), which may influence the level of consistency with which participants report them across tests. Specifically, in accordance with the second hypothesis, older adults exhibited lower consistency in true memories: 81% of initial true memories in older adults were reported consistently across tests, compared to 91% in young adults. This finding suggests that young adults tend to report true memories of witnessed events consistently and convincingly, whereas older adults show greater inconsistencies across tests. The reliability and consistency of memory reports from special witnesses, such as children and older adults, is a critical issue in legal settings. While prior studies have mainly focused on children' memory consistency, our investigation into older adults offers a novel contribution to the field (Henkel 2014a). The finding that older adults' initial true memories are more likely to become unreliable and self‐contradictory during repeated questioning raises special legal concerns. This phenomenon may be attributed to older adults' impaired memory distinctiveness. Given that memories that are well differentiated between sources and enriched with vivid details are more likely to be consistently reported (Price et al. 2016), our findings suggest that even when older adults successfully reject misinformation and report true memories, their memory distinctiveness remains lower than that of younger adults. This finding aligns with previous research showing that older adults' true memories often lack detail and are less differentiated (e.g., Koen et al. 2020). These results highlight that aging affects not only the quantity but also the quality of memory reports for original information.

Contrary to the second hypothesis, young and older adults showed similar consistency of false memories. According to the misrecollection account, older adults' false memories are often accompanied by high confidence and vividness (Dodson and Krueger 2006; Karpel et al. 2001; Mitchell and Johnson 2009). However, our finding suggests that these stronger subjective feelings did not necessarily lead to higher consistency of false memories across tests. One possible explanation is that the retrieval support provided in Test 2 effectively inhibited the sustainment of initial false memories in both young and older adults. Specifically, both groups may have benefited equally from the re‐presentation of the original image and context, which helped them reinstate memories of original details and reduce their tendency to report false memories again (Danckert and Craik 2013; Badham et al. 2016; Craik and Schloerscheidt 2011). The use of memory anchors or contextual cues in Test 2 can also be applied in legal practices. This may involve reintroducing elements of the original event, such as the appearance of key figures, objects, or surroundings (Guerin et al. 2012), the chronological order (Diamond and Levine 2020), and its underlying structure (goals, intentions, etc.) (Zacks 2020). These strategies may help older adults retrieve original information and mitigate the persistence of false memories across tests.

### 4.3 Older Adults Showed Less Reduction in False Memories With Increased Attentional Salience Than Young Adults

Consistent with the third hypothesis, although both groups reduced false memories as the attentional salience of original information increased, older adults' false memories decreased less drastically than those of young adults, leading to more pronounced age differences on high‐salience items. This finding illustrates how age differences in false memories manifest across items encoded to varying degrees and addresses previous age‐related disparities. It also provides novel insights into the distinct cognitive mechanisms underlying misinformation susceptibility in young and older adults. According to the original memory impairment hypothesis, there is a direct relationship between successful encoding of original information and subsequent reports of false memories (McCloskey and Zaragoza 1985; Okado and Stark 2005). However, by including nearly one hundred misleading items, we quantified that while encoding‐related attentional differences accounted for nearly half of false memory variations in young adults, it explained only a tenth in older adults. This suggests that encoding failures play a more prominent role in shaping false memories in young adults, whereas their influence is lesser in older adults.

Fuzzy trace theory posits two explanations for the weaker relationship between attention and false memories in older adults: (1) they struggle to engage sufficient attentional resources to form verbatim traces due to declines in information processing abilities (Greene and Naveh‐Benjamin 2024; Abadie and Guette 2024), and (2) despite successful encoding, their verbatim traces are more vulnerable to compromise from misinformation interference (Radvansky et al. 2005; Mitchell and Johnson 2009; Bulevich and Thomas 2012). Our findings favor the second explanation, as both age groups were equally effective at allocating attention to encode verbatim traces and reduce memory errors in the control condition without misinformation. This suggests that older adults' heightened susceptibility to false memories is not solely due to general difficulties in the formation of verbatim traces, but rather a consequence of inhibition, monitoring, and discrimination failures in the presence of misinformation interference. For example, the source monitoring theory posits that older adults struggle to exert sufficient cognitive control to carefully distinguish original and misleading details (Meade et al. 2012; Mitchell and Johnson 2009) and to adjust their responses based on mnemonic information (Bulevich and Thomas 2012; Fandakova et al. 2018). These retrieval insufficiencies caused by misinformation interference may prevent older adults from leveraging the enhanced verbatim traces of high‐salience items to reduce false memories. These findings can further inform future interventions to reduce false memories and suggest that such interventions should be tailored to each age group: while enhancing encoding strategies may be effective for young adults, improving retrieval monitoring processes may be more beneficial for older adults.

### 4.4 Attentional Salience Influenced Consistency of True Memory but Not False Memory in Young and Older Adults

Consistent with the fourth hypothesis, attentional salience of original details improved true memory consistency. That is, the degree of processing during the encoding of original details affects not only the incidence of true memory reports but also the quality of memory representations formed in both age groups. A thorough encoding of these image details may lead to highly specific and unique verbatim memory traces (Greene and Naveh‐Benjamin 2023b). This study extends this view by showing that these verbatim memory traces can further help both young and older adults maintain true memories across tests with different retrieval formats. However, contrary to the fourth hypothesis, no significant interaction between age and attentional salience was found. One possible explanation is that there is a ceiling effect on the consistency of true memory. Another possible explanation is that the retrieval demands were reduced in Test 2 because the re‐presentation of the original image enabled older adults to rely on their relatively intact recognition abilities to report true memories consistently across tests (Fraundorf et al. 2019).

In line with the fourth hypothesis, our study revealed no attention effect on false memory consistency. Previous studies have shown that the consistency of false memory reports relies on a firmly held false recollection that misinformation occurred (Wyler and Oswald 2016). Such false memories are not easily erased. Previous research has found that false memories persist even after the misinformation has been retracted (Ecker et al. 2011). This study provides novel evidence that strong encoding of highly salient original details also fails to reduce the consistent reports of these stubborn false memories. Instead, these convincing false memories may be more closely related to the encoding of misinformation and its erroneous incorporation into the original context (Doss et al. 2016).

### 4.5 Contributions and Limitations

This study examined age‐related differences in the accuracy and consistency of memory reports across two different tests and assessed the role of attentional salience of original information in influencing these differences. The study made three main contributions. First, this study clearly demonstrated that attentional differences during the encoding of original details affected age‐related differences in false memories. Older adults failed to reduce false memories to the level of their younger counterparts as attentional salience increased. Second, this study went beyond the static examination of age‐related memory patterns in a single memory test and investigated memory consistency across successive retrieval sessions. The findings revealed that older adults demonstrated lower consistency in true memories but not in false memories. This has practical implications for highlighting the potential unreliability of older witnesses' true memory reports under repeated questioning. This finding also deepens our understanding of the reduced distinctiveness of older adults' true memories. Third, this study illustrated the impact of attentional salience on both memory accuracy and consistency. Of note, its effect on memory consistency followed distinct patterns for true and false memories. Specifically, while attentional salience enhanced true memory consistency, it had minimal impact on false memory consistency for both young and older adults. These findings highlight the importance of strong encoding of highly salient original details in preserving true memories across tests and the weaker relationship between original encoding and subsequent consistency of false memories.

This study had several limitations. First, memory consistency was examined between two tests administered consecutively. Future research could explore how young and older adults perform across multiple tests spaced over longer intervals, such as several weeks (Odinot et al. 2012). Second, both tests used in this study were recognition‐based. While this format provides a standardized measure of memory consistency and has been commonly adopted (Henkel 2014b; Zhu et al. 2012), it may not fully capture real‐world recall situations and may overlook spontaneous memory responses, such as omission errors or reminiscence. Future research could employ recall‐based paradigms to provide a more comprehensive understanding of memory inconsistencies across tests (Price et al. 2016). Third, the link between memory consistency and memory quality was not directly evaluated in our experiment. Future research could combine successive memory tests with direct quality measures, such as confidence ratings, to better clarify the mechanisms underlying memory consistency in young and older adults (Odinot et al. 2012). Fourth, older adults with intact cognitive abilities (i.e., MMSE score above 24) were included in this study. However, older adults with impaired cognitive functioning are often more vulnerable to misinformation‐induced false memories (Roediger and Geraci 2007; Brassil et al. 2024). The extent to which they can benefit from strong encoding of salient original details to reduce false memories and improve the consistency of true memory reports requires further investigation.

## 5 Conclusions

The present study aimed to investigate how young and older adults responded to misleading information and how these responses affected the accuracy and consistency of their memory reports across two successive tests. This study also explored how attentional salience of original details influenced age differences in memory accuracy and consistency. First, while older adults were more susceptible to misinformation overall, our results revealed that these age differences were exacerbated as attentional salience of original information increased, because older adults were less able than their younger counterparts to reduce false memories despite increased attention. Second, in addition to age differences in memory accuracy, young and older adults also differed in the consistency of their memory reports across tests. Specifically, older adults exhibited lower consistency of true memories and similar consistency of false memories. These findings indicate a reduced distinctiveness in older adults' original memories and raise concerns about their vulnerability to unstable true memory reports during repeated questioning. Third, attentional salience of original details affected both the accuracy and consistency of memory reports. However, its effect on the consistency of true and false memories followed distinct patterns: increased attentional salience of original details enhanced true memory consistency, but had minimal impact on false memory consistency. This study enhances our understanding of older adults' vulnerability to false and inconsistent memories across tests and offers practical implications for improving the reliability of their testimonies.

## Conflicts of Interest

The authors declare no conflicts of interest.

## Figures and Tables

**FIGURE A2:**
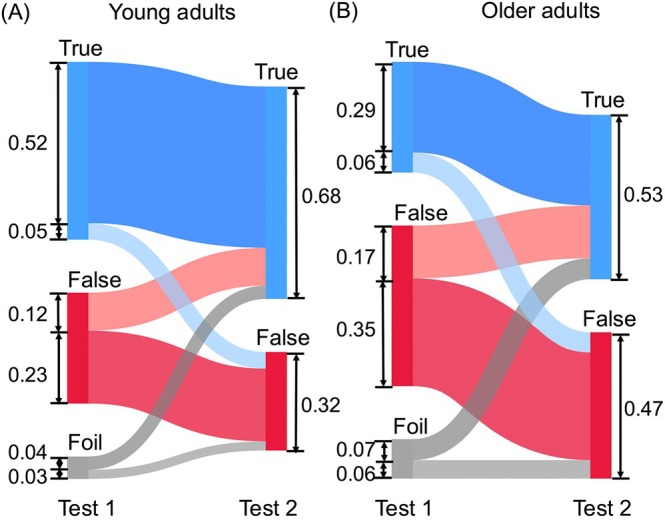
Sankey diagram illustrating memory responses across tests in young adults (A) and older adults (B). The number beside each connecting arc represents the mean endorsement rate for each memory type in each test. The size of each node and the width of each arc are proportional to the mean endorsement rate.

## Data Availability

The data that support the findings of this study are available from the corresponding author upon reasonable request.

## Appendix A

### Young and Older Adults' Ratings of Attentional Salience of Original Images

#### Participants

Sixteen independent raters participated in the pilot study to evaluate the attentional salience of original details. The sample included eight young adults (4 females and 4 males, mean age = 21 ± 1 years) and eight older adults (4 females and 4 males, mean age = 70 ± 3 years). Both young and older adults were recruited to ensure variability in the sample and to examine potential rating disparities between the two groups (Koo and Li 2016). The sample size was selected in accordance with those of previous studies (2 and 13 raters; e.g., Payne et al. 2006; Wyler and Oswald 2016).

#### The Rating Procedure

Participants initially viewed images from six events sequentially, following the same procedure as the original‐event stage in the main experiment. Subsequently, each image for misleading and control items was shown alongside the corresponding question tested, and participants were asked to assign a score from one to six to indicate the extent to which the probed image details had attracted their attention during the initial viewing period. A score of one represented a complete lack of attention, whereas a score of six represented very focused attention toward the detail. No misinformation was introduced during the rating procedure.

#### Evaluation of Group Differences in Attention Ratings and Interrater Reliability

A differential interrater reliability (IRR) analysis and a Kolmogorov–Smirnov test were performed to reveal any rating disparities between young and older adults (Bartoš et al. 2020; Martinkova et al. 2018; Fairfield et al. 2017). First, the differential IRR analysis was used to assess the consistency of ratings across groups. By decomposing rating variance into components attributable to ratees and random errors, this method identifies group‐level differences in rating patterns or variabilities (Bartoš et al. 2020; Martinkova et al. 2018). Specifically, the following linear mixed model was built to decompose variances by group via the lme4 package with REML fitting (Bates et al. 2014): y ~ 1 + group + (0 + group|item). In accordance with Shrout and Fleiss (1979), the IRR was defined as the proportion of the ratee variance to the overall variance. Therefore, the IRR for each group was calculated as the variance of the items for each group divided by the sum of the variance of the items for each group and the residual variance. Differences in the IRR between groups were then calculated. The results were bootstrapped 1000 times to obtain the standard errors and differential IRR estimates with confidence intervals (CIs). The results showed that the differential IRR had 95% CI values ranging from −0.006 to 0.105, which indicated that both groups adhered to consistent rating standards and demonstrated consensus in rating the attentional salience of original details.

Second, a Kolmogorov–Smirnov test was conducted to compare the rating distributions between groups (Fairfield et al. 2017). This nonparametric method evaluates distribution differences, such as shape and location, by comparing the empirical cumulative distributions of the two chosen samples. The test result (*D* = 0.10, *p* = 0.38) suggested that both groups exhibited similar tendencies in their perceptions and judgments of attentional salience (Figure A1).

**TABLE A1:** Means and standard deviations of endorsement rates for consistent and inconsistent memory responses to misleading items across tests among young and older adults.

Age group	True ➔ True	True ➔ False	False ➔ False	False ➔ True	Foil ➔ True	Foil ➔ False
Young	0.52 (0.10)	0.05 (0.03)	0.23 (0.10)	0.12 (0.04)	0.04 (0.02)	0.03 (0.02)
Older	0.29 (0.10)	0.06 (0.03)	0.35 (0.10)	0.17 (0.04)	0.07 (0.03)	0.06 (0.02)

*Note*: Endorsement rates were calculated by dividing the number of endorsements by the total number of misleading items. In Test 1, true memories included both consistent (true‐to‐true) and inconsistent (true‐to‐false) responses. Similarly, false memories included consistent (false‐to‐false) and inconsistent (false‐to‐true) responses. Foil memories included foil‐to‐true and foil‐to‐false responses.

Since no group differences were observed, we treated the 16 raters as a single “class” and calculated the intraclass correlation (ICC) among them. The ICC estimate and its confidence intervals were calculated on the basis of Shrout and Fleiss (1979) via the psych R package with a mean‐rating (*k* = 16), consistency, 2‐way mixed‐effects model. The ICC (2, *k*) was 0.91 (95% CI [0.89–0.93]), indicating excellent interrater reliability according to customary standards (Koo and Li 2016).

## Appendix B

### Age Differences in Memory Accuracy in Test 1 After Controlling for Education

The original 2 (age group: young and older adults) × 4 (memory type: true, false, foil, and correct) ANOVA was extended to include years of education as a covariate. Results revealed a significant interaction between age group and memory type, *F*(3,171) = 28.20, *p* < 0.001. Compared with young adults, older adults had lower rates of true memories (*t*(57) = −7.01, *p* < 0.001), higher rates of false memories (*t*(57) = 5.29, *p* < 0.001), and higher rates of foil memories (*t*(57) = 3.50, *p <* 0.001). However, both groups showed similar rates of correct memories after controlling for education (*t*(57) = −1.11, *p* = 0.274).

The 2 (age group: young and older adults) × 2 (memory type: false and incorrect) ANOVA, with education as a covariate, revealed a significant interaction (*F*(1,57) = 18.07, *p* < 0.001), and significant main effects of age group (*F*(1,57) = 29.74, *p* < 0.001) and memory type (*F*(1,57) = 421.37, *p* < 0.001), respectively. Older adults exhibited greater susceptibility to misinformation than young adults after controlling for their general memory decline (difference between false memory and incorrect memory, older adults: *M*_diff_ = 0.37, *t*(57) = 16.02, *p <* 0.001; young adults: *M*_diff_ = 0.22, *t*(57) = 9.33, *p* < 0.001).

## Appendix C

### Age Differences in Memory Accuracy in Test 2

An independent *t*‐test was conducted to compare young and older adults on the endorsement rate of false memories in Test 2. Results revealed that older adults had higher rates of false memories than young adults (older adults: *M* = 0.47, SD = 0.10, young adults: *M* = 0.32, SD = 0.10; *t*(58) = 5.95, *p* < 0.001, Cohen's *d* = 1.54).

## Appendix D

### The Gains and Losses in Memory Accuracy for Misleading Items Across Tests

Memory responses for misleading items in Test 1 (true, false and foil) were fully crossed with those in Test 2 (true and false) to generate six memory types in total. The endorsement rates of these memory types in young and older adults are reported in Table A1 and Figure A2.

To investigate memory accuracy across repeated testing and to assess whether Test 2 improved memory accuracy for misleading items, whether gains in memory accuracy exceeded losses across tests was compared in young and older adults. Memory gains and losses were defined in two ways. In the first definition, memory gains referred to the endorsement rate of false‐to‐true memories across tests, while losses referred to true‐to‐false memories across tests. The second definition accounted for foil memory changes, with gains calculated as the sum of endorsement rates for false‐to‐true and foil‐to‐true memories, and losses as the sum of true‐to‐false and foil‐to‐false memories. For each definition, a 2 (age group: young and older adults) × 2 (memory type: gains and losses) mixed‐effects ANOVA was performed.

For the first definition, results revealed a significant interaction effect (*F*(1,58) = 7.40, *p* = 0.009, *η*_*p*_^2^ = 0.11), and significant main effects of age group (*F*(1,58) = 19.36, *p* < 0.001, *η*_*p*_^2^ = 0.25) and memory type (*F*(1,58) = 168.51, *p <* 0.001, *η*_*p*_^2^ = 0.74), respectively. These findings suggest that older adults experienced more memory changes across tests than young adults, and both groups corrected more false memories than they negated true memories when tested again with retrieval cues. However, the absolute net gain was greater for older adults than for young adults (difference between memory gains and losses, older adults: *M*_diff_ = 0.11, *t*(58) = 11.10, *p* < 0.001, Cohen's *d* = 2.03; young adults: *M*_diff_ = 0.07, *t*(58) = 7.26, *p <* 0.001, Cohen's *d* = 1.32).

For the second definition, the results replicated previous findings of significant main effects of age group and memory type (*F*(1,58) = 40.67, *p* < 0.001, *η*_*p*_^2^ = 0.41; *F*(1,58) = 143.98, *p* < 0.001, *η*_*p*_^2^ = 0.71). Older adults experienced more memory changes than young adults, and both groups showed more gains than losses (older adults: gains = 0.24, losses = 0.12, *t*(58) = 9.89, *p <* 0.001, Cohen's *d* = 1.85; young adults: gains = 0.16, losses = vs. 0.08, *t*(58) = 7.08, *p <* 0.001, Cohen's *d* = 1.26). The interaction effect was marginally significant, *F*(1,58) = 3.93, *p* = 0.052, *η*_*p*_^2^ = 0.06.

## Appendix E

### Age Differences in Memory Consistency After Controlling for Education

The 2 (age group: young and older adults) × 2 (memory type: true and false) ANOVA on consistency ratios, with education as a covariate, revealed a significant interaction effect (*F*(1,57) = 4.96, *p* = 0.030). Post hoc analysis revealed that older adults had lower consistency ratios for true memories compared to young adults (older adults: *M*_true_ = 0.81, young adults: *M*_true_ = 0.90, *t*(57) = −3.63, *p <* 0.001), while there was no significant difference in consistency ratios for false memories between the two groups (older adults: *M*_false_ = 0.66, young adults: *M*_false_ = 0.64, *t*(57) = 0.39, *p =* 0.697). The main effect of memory type was significant, *F*(1,57) = 121.15, *p* < 0.001. The main effect of age group was marginally significant, *F*(1,57) = 2.98, *p* = 0.090.

## Appendix F

### The Effect of Attentional Salience on Memory Accuracy in Test 1 Among Young and Older Adults With Matched Education

To rule out the possibility that the effects of age and attentional salience on memory accuracy in Test 1 were influenced by education differences between young and older adults, only those with more than 12 years of education (i.e., those who attended college) were included in this analysis. All young adults and 14 older adults met this criterion. A Welch *t*‐test comparing this group of young and older adults on years of education showed no significant differences (*M*_young_ = 16.77, *M*_old_ = 16.21, *t*(38) = 1.74, *p =* 0.089).

Regression models were constructed as in the main analyses, except that the proportions of participants who reported true memories, false memories, and incorrect memories were based on 30 young adults and 14 older adults in each age group. Results found a significant interaction for true memories (*β* = −0.04, *t* = −2.25, *p* = 0.026, *p*_FDR_ = 0.038), showing that the attentional salience of original details increased true memories in both age groups, though the effect was weaker in older adults (older adults: *β* = 0.08, *t* = 5.85, *p* < 0.001; young adults: *β* = 0.12, *t* = 10.05, *p* < 0.001). A significant interaction was also observed for false memories (*β* = 0.06, *t* = 3.12, *p* = 0.002, *p*_FDR_ = 0.006), showing that the attentional salience reduced false memories in both age groups, though the effect was weaker in older adults (older adults: *β* = −0.04, *t* = −2.30, *p* = 0.024; young adults: *β* = −0.09, *t* = −8.42, *p* < 0.001). The regression on incorrect memories showed an insignificant interaction between attentional salience and age group (*β* = 0.01, *t* = 0.84, *p* = 0.401, *p*_FDR_ = 0.401), and the magnitude of the negative relationship between attentional salience and incorrect memories was similar across age groups (older adults: *β* = −0.02, *t* = −1.91, *p* = 0.062; young adults: *β* = −0.03, *t* = −3.46, *p* = 0.001).

## Appendix G

### The Effects of Age and Attentional Salience on Memory Accuracy in Test 2

Linear regression models were constructed, as in the main analyses, to predict the proportion of participants who reported false memories for each misleading item in Test 2. The results mirrored those from Test 1, showing a significant interaction between attentional salience and age group (*p* = 0.011), with older adults demonstrating a weaker relationship between attentional salience of original details and false memories than young adults (older adults: *β* = −0.05, *t* = −5.71, *p* < 0.001; young adults: *β* = −0.09, *t* = −8.34, *p <* 0.001).

## Appendix H

### The Effect of Attentional Salience on Memory Consistency Among Young and Older Adults With Matched Education

True and false memory consistency ratios based on 30 young adults and 14 older adults with matched education were predicted by attentional salience, age group, and their interaction. Results showed significant main effects of age group and attentional salience on true memory consistency (*ps* < 0.005), with no significant interaction (*p* = 0.808). Attentional salience of original details increased true memory consistency in both groups (older adults: *β* = 0.05, *t* = 2.58, *p* = 0.011; young adults: *β* = 0.06, *t* = 5.79, *p* < 0.001). For false memory consistency, no significant effects were observed for attentional salience, age group, or their interaction (*ps* > 0.622).
